# The Effects of Physical Education on Motor Competence in Children and Adolescents: A Systematic Review and Meta-Analysis

**DOI:** 10.3390/sports8060088

**Published:** 2020-06-15

**Authors:** Håvard Lorås

**Affiliations:** Department of Teacher Education, Faculty of Social and Educational Sciences, NTNU—Norwegian University of Science and Technology, 7491 Trondheim, Norway; havard.loras@ntnu.no

**Keywords:** motor skills, teaching, motor intervention, motor behavior, physical activity

## Abstract

Appropriate levels of motor competence are an integrated part of individuals’ health-related fitness, and physical education is proposed as an important context for developing a broad range of motor skills. The aim of the current study was to apply meta-analyses to assess the effectiveness of curriculum-based physical education on the development of the overall motor competence of children and adolescents. Studies were located by searching seven databases and included according to predefined criteria. Random effects models using the standardized effect size (Hedges’ *g*) were used to aggregate results, including an examination of heterogeneity and inconsistency. The meta-analysis included 20 studies, and a total of 38 effect sizes were calculated. A statistically significant improvement in motor competence following curriculum-based physical education compared to active control groups was observed in children and adolescents (*g* = −0.69, 95% CI −0.91 to −0.46, n = 23). Participants’ ages, total time for physical education intervention, and type of motor competence assessment did not appear to be statistically significant moderators of effect size. Physical education with various curricula can, therefore, increase overall motor competence in children and adolescents.

## 1. Introduction

The level of motor competence amongst children and adolescents has been systematically linked in recent decades to numerous important health-related factors, such as cardiorespiratory and musculoskeletal fitness [1,2] and activity levels/participation in organized physical pursuits [3]. Thus, motor competence impacts upon children’s physical, social, and cognitive development [4]. In the scientific literature, motor competence is generally a global term used to reflect various other terminologies that have been applied previously (i.e., motor proficiency, motor performance, fundamental movement/motor skills, motor ability, and motor coordination) to describe goal-directed human movement [5]. For instance, general motor competence has been considered synonymous with fundamental motor/movement skills that include locomotor and object control skills [6]. A more specific and related definition originated with Henderson and Sugden (1992): “A person’s ability to execute different motor acts, including coordination of fine and gross motor skills that are necessary to manage everyday tasks” [7].

Low levels of motor competence have been reported for both children and adolescents in many countries [8,9,10,11]. In order to increase these levels among children and youth who are potentially missing out on developing motor behaviors that are important for normal growth and development, practice and instruction are needed to facilitate motor development [12]. Indeed, research has consistently demonstrated that scheduled programs that are developmentally and age appropriate (for children or adolescents), activity-based, task-specific, and/or have a high level of autonomy are efficient interventional methods for improving various aspects of motor competence [13,14,15]. Furthermore, schools have been identified as a key setting, with effective interventions for reaching out across developmental periods [16,17].

Physical education as an integrated component of the curriculum, and thus holds great potential for maintaining and developing children and adolescents’ motor competence. In many countries, physical education classes are mandatory at both the primary and secondary levels, and for some sedentary children and adolescents, such classes may represent the only context in which they experience physical activity and motor challenges. Indeed, guidelines from national education ministries typically state that, through physical education, pupils shall experience motor learning and development by conducting a wide range of activities (e.g., swimming, team and individual sports, play and dance), indoors and outdoors. An example is the Australian National Curriculum for Health and Physical Education, which states: “The content enables students to develop and practice fundamental movement skills through active play and structured movement activities” [18]. Similarly, England’s National Physical Education Curriculum states: “… Pupils should develop fundamental movement skills…” [19], and for Singapore, the national curriculum states: “… The school’s physical education program is a primary contributor to building a strong foundation for the development of broad-based physical competencies …” [20] Furthermore, contemporary theoretical approaches and models for teaching and instructing physical education classes for both children and adolescents advance key concepts for supporting and fostering the learning of movement skills through the organization of the learning environment and the effective and efficient use of practice time [21,22].

Physical education, as an important context for developing motor competence, also holds great potential for influencing academic development and learning in a broader sense. Fine motor skills (in particular) have emerged as an important predictor of learning in the early years of schooling. Empirical evidence from large longitudinal data sets across Britain and the US suggests that initial fine motor skills is systematically linked to later academic achievement (math, reading and science). Such data clearly demonstrates that level of motor competence should be an integrated part of school readiness indicators [23]. Independent investigations have provided further evidence for such a motor-cognitive link, as fine motor skills in kindergarten have been shown to predict levels of reading in grade 1 [24], and grade 1 levels of fine motor integration significantly predict math ability [25].

The effects of physical education on the components of motor competence have been researched and debated since at least the 1960s. Whittle’s study (1961) reported the effects of elementary-school physical education on aspects of development (physical, motor and personality), claiming benefits for the pupils in the study [26], and Smith (1982) demonstrated effects on movement skill levels in third-grade children following a physical education program [27]. A marked increase in published studies on physical education, physical activity and motor competence (spiked, perhaps, by the global burden of obesity in children and adolescents [28]) has emerged over the past two decades. In previous systematic reviews with meta-analyses, however, reviewed studies have focused on the effects of various motor interventions on fundamental motor skills (FMS). Van Capelle et al. [29] and Wick et al. [30], for example, reported that teacher-led interventions improved FMS in children between 3 and 5 years of age, and Logan et al. [13] and Morgan et al. [14] found that developmentally appropriate motor interventions improved FMS in children and adolescents. None of these reviews included studies on physical education, however. In a recent meta-analysis by Jimenez-Diaz et al. [31], the authors reported statistically significant improvements in motor competence following physical education classes (standardized mean effect size = 0.52; 95% CI, 0.08 to 0.97). A close examination of the included studies, however, reveals that the data originated from various sub-samples (e.g., normally developing children vs. clinical samples) and different ages (early preschool vs. late adolescence/young adults) that were pooled together. Furthermore, several of the studies did not provide sufficient background information to be classified as occurring in a school-based physical education setting. Additionally, the forest plot for physical education classes indicated a substantial heterogeneity of findings, ranging from a substantial positive effect size (4.89) and a zero effect size (0.04) to a moderately negative effect size (−0.59). Such findings warrant a closer examination of potential moderators of the effect sizes emerging from studies on the results of physical education on motor competence.

Based on the presented considerations, the aim of the current review was to apply aggregate meta-analysis procedures to assess the effectiveness of curriculum-based physical education for developing motor competence in children and adolescents. The included studies reported data on physical education interventions conducted with groups/classes of pupils that were compared to control groups. The overall effect was further examined in relation to the average age of participants (children and adolescents), type of motor competence assessment, and total duration of intervention as possible moderating factors.

## 2. Materials and Methods

This meta-analysis followed the general recommendations of the Preferred Reporting Items for Systematic Reviews and Meta-Analyses (PRISMA) statement [32].

### 2.1. Study Eligibility Criteria

To be included in this meta-analysis, studies had to be in line with the following criteria: (1) intervention trials that included physical education classes and control groups; physical education classes should be characterized by teacher-led activities that included instructions following the general guidelines (standard curriculum) of the school, city, or country; (2) participants were normally developing preschool/school-aged children and adolescents of any sex and motor skill level; (3) assessment of overall motor competence as a dependent variable; (4) data presented for the computation of a standardized mean difference effect size (means, standard deviations, and sample sizes); (5) studies published in a peer-reviewed journal; and (6) studies published in the English language. There were no restrictions on year of publication. For the current meta-analysis, overall motor competence was considered a participant’s summated performance in forms of goal-directed motor tasks that predominantly require coordination and control of the human body, including fundamental movement/motor skills (locomotor and object control) and balance tasks.

### 2.2. Data Sources

**Figure 1:**
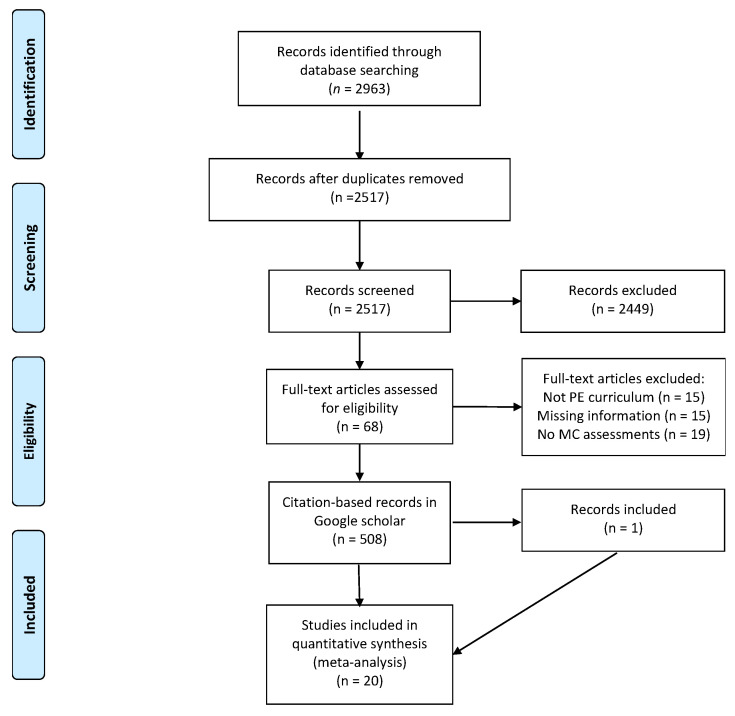
Flow diagram and literature search process.

A description-based literature search for potentially eligible studies published up to 14 February 2020, was conducted via computerized searches using the following seven electronic databases: EBSCOhost (including Psychology and Behavioral Sciences Collection, Education Source, ERIC, SPORTDiscus) and Ovid (including PsycINFO, MEDLINE and Embase). These sources were selected in order to provide broad coverage of journals from both the social and life sciences, e.g., Education Source is the world’s largest bibliographic database designated to educational research, and MEDLINE, as a bibliographic database of life sciences, contains >26 million records. The following general search string was used: (Physical education AND (motor performance OR motor skill OR motor ability OR motor competence) AND (children OR adolescents) NOT animal) without any special limits. In addition to electronic database searches, the reference lists of retrieved articles were reviewed, as well as the reference lists of previous systematic reviews. Furthermore, based on the results of the description-based searches, citation-based searches [33] were conducted using Google Scholar. All English-language articles citing each of the previously identified articles were examined, in order to identify additional potential articles to include. The articles identified had been cited by others a total of 508 times, and one of these articles was ultimately included in the present meta-analysis. A flow diagram of the search process can be found in Figure 1.

### 2.3. Study Selection and Data Abstraction

Prior to data abstraction, a codebook was created in Microsoft Excel (Excel 2016; Microsoft, Redmond, WA, USA). The general items coded included the sample size, participants’ mean age, physical education characteristics and assessment of overall motor competence. Given that the total amount of time for physical education classes can vary substantially across studies, a summated physical education exposure (in hours) was also estimated based upon provided information on the duration of PE sessions, the number of sessions per week, and the duration of the study period (months).

### 2.4. Calculation of Effect Size

The primary outcome (overall motor competence) for the current meta-analysis was the standardized mean difference effect size (Hedges’ *g*, [34]) calculated from pretest-posttest within-group means and standard deviations reported in the included studies. When a study reported information for different experimental groups, all possible effect sizes were calculated. All effect sizes were checked individually, and a negative effect size signifies that overall motor competence improved following physical education.

### 2.5. Pooling of Effect Size

A random effects model (DerSimonian and Laird method) was used to pool effect sizes [35]. For studies that included more than one effect size (i.e., multiple outcomes for overall motor competence) for the results, these were summated according to Borenstein et al. [36], so that only one effect size represented each study. Effect sizes for different intervention groups were retained, however, as they provide data on specific comparisons of physical education intervention and control groups. All analyses were conducted using Stata 16.1 (StataCorp, College Station, TX, USA). Nonoverlapping 95% CIs were considered statistically significant.

### 2.6. Heterogeneity, Inconsistency, and Small-Study Effects

Heterogeneity across the analyzed reports was measured using Cochran’s Q test, whereas inconsistency was assessed using the *I*^2^ statistic [37]. Significance for Q was set at *p* ≤ 0.10. *I*^2^ values < 25% were considered to represent very low; 25% to <50% to represent low; 50% to <75% to represent moderate, and 75% or more to represent large amounts of inconsistency. To assess for the possible presence of small-study effects (publication bias, language bias, citation bias, and time lag bias), a funnel plot and Egger’s regression test were applied [38].

### 2.7. Moderator Variables

Moderator variables, determined a priori, included age and duration of the physical education intervention (total number of hours) and assessment of motor competence. Moderator analyses were accomplished using meta-regression and an analysis of variance-like models for meta-analysis with Stata. A two-sided alpha level ≤ 0.05 was considered statistically significant.

## 3. Results

### 3.1. Overview of Included Studies

A total of 20 studies published between 2002 and 2020 were included in the meta-analysis (see Table 1 for the main characteristics). These studies included 38 effect sizes, representing 4009 participants between 3 and 13 years of age. According to international classification of developmental periods [39], 12 studies were conducted on children and 8 studies on adolescents. Total time (exposure) of physical education classes in the interventions ranged from 4–73 h, and sample size varied from 40–509 participants. Physical education interventions consisted of a wide range of physical education curricula (e.g., gymnastics, physical literacy, fundamental motor/movement skills, health/fitness), and comparison groups (active) in most of the included studies (85%), which followed the regular/traditional curriculum in their physical education classes. Furthermore, eight studies (see Table 1) included a version of the Test of Gross Motor Development (TGMD) for the overall assessment of motor competence. TGMD is made up of 12 skills divided into two subtests: (1) Locomotor: Run, gallop, hop, leap, horizontal jump, slide and (2) Object Control: Striking a stationary ball, stationary dribble, kick, catch, overhand throw, and underhand roll [40]. Four studies applied the Körper Koordinationstest Für Kinder (KTK), which consists of four tasks: (1) walking backward along a balance beam of decreasing width: (2) two-legged jumping from side to side for 15 s; (3) moving sideways on wooden boards for 20 s; and (4) hopping for height [41]. Further on, eight studies adopted assessments based upon concepts of fundamental movement/motor skills [42], which consisted of variations of jumping, running, rolling, crawling, throwing, balancing, catching and kicking. The remaining studies based their assessments upon concepts of physical literacy [43] and the applied testing of locomotor, balancing, running, jumping and object-control tasks.

### 3.2. Effect of Physical Education on Overall Motor Competence

**Figure 2:**
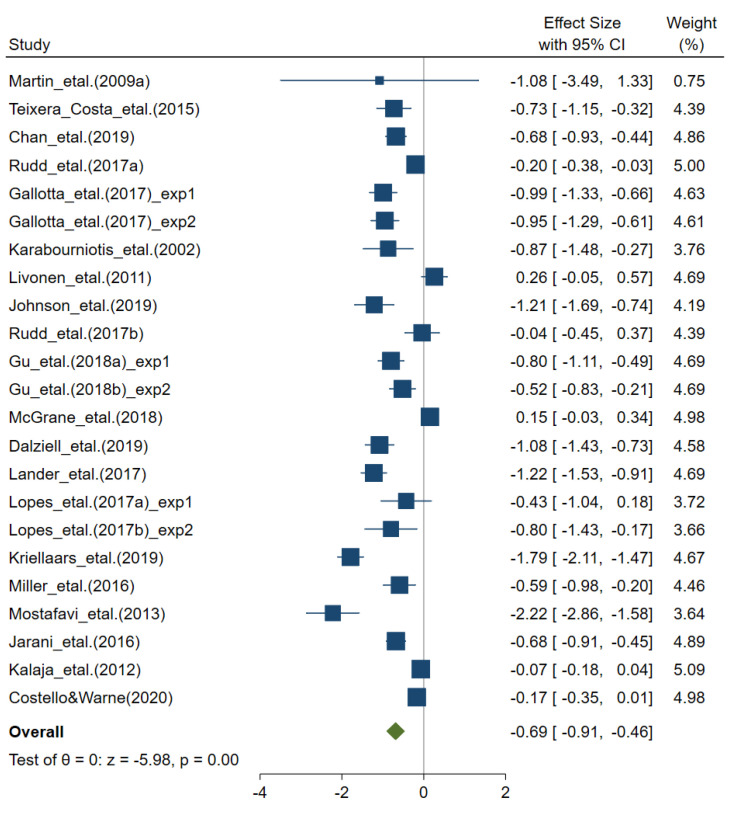
Forest plot for effect of curriculum-based physical education on motor competence. Note: One representative effect size was coded for overall motor competence assessed.

As depicted in Figure 2, the 20 included studies with data from 23 physical education intervention vs. control group comparisons indicated statistically significant positive effect sizes of physical education classes on overall motor competence (*g* = −0.69, 95% CI −0.91 to −0.46, *n* = 23, *Q* = 303.01, *p* < 0.001, *I*^2^ = 92.74%). In a subsequent analysis, the studies adopting the TGMD test (*n* = 8) were compared to studies including other types of assessments. The latter analysis was conducted as a proxy for effects of types of motor competence assessments. The effect size was statistically significant and similar, however, in subgroup studies with the TGMD (*g* = −0.67, 95% CI −1.01 to −0.25, *n* = 8, *Q* = 87.16, *p* < 0.001, *I*^2^ = 91.97%) compared to other assessments (*g* = −0.69, 95% CI −0.91 to −0.46, *n* = 15, *Q* = 208.94, *p* < 0.001, *I*^2^ = 93.30%). This suggests a similar effect of physical education classes on overall gross motor skills compared to other types of assessments of motor competence, based upon concepts of fundamental movement/motor skills and physical literacy. In a further meta-regression analysis, age and total time in physical education classes (exposure) were examined as potential effect size moderators. This latter analysis indicated no significant association between the mean age of participants and total amount (number of hours) of physical education classes in the interventions (*p* > 0.05 for both). In a further examination of age as a possible moderator, studies with children (<10 years old) and adolescents (≥10 years old) were analyzed separately. Subgroup analysis indicated the statistically significant and similar effect size of physical education on overall motor competence in both developmental groups: Children: *g* = −0.59, 95% CI −0.87 to −0.33, *n* = 13, *Q* = 90.79, *p* < 0.001, *I*^2^ = 86.78% and adolescents: *g* = −0.78, 95% CI −1.17 to −0.38, *n* = 10, *Q* = 212.22, *p* < 0.001, *I*^2^ = 95.76%.

### 3.3. Heterogenity and Publication Bias

Potential small-study effects were not observed, as indicated by a lack of funnel plot asymmetry and a non-significant Egger regression test (*t* = −1.17, *df* = 372, *p* = 0.26). *I*^2^ values above 75% and significant Q statistic were found, however, which indicates high heterogeneity and the inconsistency of effect sizes.

## 4. Discussion

The primary aim of this meta-analysis was to assess the effect of various curriculum-based physical education interventions on overall motor competence in children and adolescents. The results of the aggregate meta-analysis suggest that participation in a physical education class has a positive effect on the development of motor competence in both developmental periods. In the current study, type of assessment, age of participants, and time allocated to physical education intervention did not appear as statistically significant moderators of the significant effect of physical education on motor competence.

The overall significant effect size depicted in Figure 1 appears to be, by any standard, at such a level that it signifies a clear effect of curriculum-based physical education on the development of motor competence. Although the judgment of magnitude of effects is not a straightforward scientific exercise [64], the effect size cut-offs, indicating a practically relevant effect given in the research literature, are usually at a lower end. For example, Ferguson [65] recommended that a minimum effect size representing a “practically” significant effect amounts to *g* ≥ 0.41, and Hattie [66] has suggested that effect sizes at *g* ≥ 0.40 represent a “hinge-point” at which interventions provide relevant outcomes for teaching and learning. Adding to the overall interpretation of the effect sizes obtained in the present meta-analysis, the 95% confidence intervals demonstrate no zero crossing for the overall effect size. This latter finding is a strong indicator that the null hypothesis (no effect of physical education on overall motor competence) should be rejected [67].

It should be acknowledged that the effect sizes presented in the current analysis are, for the most part, from studies that had an active comparison group. Thus, specific physical education curricula that include gymnastics, fundamental motor/movement skills and physical literacy were compared to what are reported to be current practices and curricula in physical education. Given that the studies included in the meta-analysis originate from 13 different countries, it is difficult to assess the specific content of the physical education conducted by participants in the comparison groups. This may explain why physical education studies have reported somewhat lower effect sizes compared to other movement programs, as these latter studies typically have a waitlist, non-active, control group [31]. It should come as no surprise that active-movement-based programs introduce substantial effects above what might be general motor-development effects observed in a non-active control group. Therefore, it appears that specific curricula with physical education practices targeted at different aspects of motor competence are effective for motor development.

Adding to the overall significant effect of physical education on motor competence seen in the intervention groups, a close examination of the data collected from active control groups further reveals that all included studies reported a positive change in overall motor competence in the active comparison groups reported to have followed standard physical education curricula. Thus, not all curriculum-based physical education programs have the same effect on the development of motor competence. The strongest effect appears to emerge from studies such as that by Johnson et al. [60], which assessed changes in motor competence (using the TGMD-3), after a complete academic year of physical education classes. The experimental group practiced fundamental motor skills (FMS) with stations based on the TGMD-3 test, and the control group was given greater autonomy using the same equipment and received no specific FMS instruction. In addition, the intervention group received physical education based on the principles of building a mastery–motivational climate. The results indicated a substantial difference in the increase of motor competence scores in favor of the experimental group [60]. In comparison, Rudd et al. [53] reported a smaller difference in the development of motor competence in their gymnastics intervention group, compared to a standard physical education curriculum comparison group comprising primarily team sports. These studies illustrate that inter-relatedness between assessments and physical education content (i.e., practicing and testing FMS), as well as the relative difference between intervention groups and comparison physical education curricula, have an impact on the reported effect sizes. Further controlled studies on the effect of physical education on motor competence should, therefore, carefully consider the types of assessments and their associations with physical education content, as well as the choice of active comparison group.

The results of the current meta-analysis, and those of previous meta-analytical studies investigating the effect of various type of interventions on motor competence, clearly suggest that structured programs are effective in influencing the development of motor competence in children and adolescents [13,14,29,30,31]. It has been claimed, however, that interventions defined under the term “movement programs” have a better and more consistent effect on motor competence compared to physical education. According to Jimenez-Diaz et al. [31], these consist of structured programs with specific and age-appropriate motor activities and teaching. At first sight, these programs are difficult to distinguish from physical education classes as, typically, they are also teacher-led and age-appropriate according to national curricula. One also needs to acknowledge that curriculum-based physical education has the potential to increase fitness levels alongside the development of motor competence [68,69]. This might explain the differences observed in the effects on motor competence between so-called movement programs and physical education, as the latter generally involves a greater variety of activities according to national curricula.

The presented results must be interpreted against the background of significant heterogeneity among the effect sizes extracted from different studies. As Higgins et al. [37] have pointed out, such meta-analytical variability across study results typically emerges from a diversity of methodological approaches. The studies included in the meta-analysis (besides the investigated effects of types of assessments, ages of participants and physical education exposure), also differed in terms of curriculum and specific content of the physical education classes. Based on empirical and theoretical work on motor development and learning [12,70], it is expected that effects on motor competence are highly specific towards the content of the physical education classes. Thus, a substantial increase in, for example, gross motor skills, can be found if the classes include similar types of gross movements/exercises that can be found in the assessment batteries. Indeed, studies that reported the most-pronounced effect sizes in the current meta-analysis were relatively close in similarity to physical education content and type of assessment [55,60,62]. Furthermore, differences in risk of bias can also relate to heterogeneity in study results included in the current meta-analysis [71]. Albeit, many checklists and scoring systems have been developed for evaluating methodological quality and risk of bias for different kind of study designs, there appears to be no checklist developed specifically for educational trials. Still, considering items related to study quality and study reporting typical for various checklists [72,73], the studies included in the current meta-analysis all specified eligibility and variations of randomization/matching, as well as similarity in motor competence measures at baseline. Furthermore, point measures, measures of variability and between-group statistical comparisons for motor competence were also reported. In addition, important intervention parameters, such as duration and frequency of sessions, were stated. Altogether, this suggests that (across studies) a certain degree of methodological rigor has been applied, which prevents, to some extent, the systematic risk of bias. As the included studies have been conducted in a naturalistic setting (as a part of the children and adolescents PE routines), some degree of trade-off between issues of validity and reliability is to be expected.

As depicted in Figure 2, the weighted mean effect size amounted to *g* = 0.69 for the effect on motor competence after curriculum-based physical education classes. This suggests that a substantial proportion of pupils (children and adolescents) might benefit from such classes, in terms of developing increased motor competence. Furthermore, it points to physical education in a school setting as an important context in terms of targeting health-related aspects, as the level of motor competence is systematically linked to a number of physiological and psychological factors [1,5]. This is further highlighted by developmental trends typically seen in industrialized societies where populations’ sedentary behaviors are increasing [74]. As substantial numbers of children and adolescents are dropping out of sports [75], have a lack of recreational areas for physical activity near their homes, and rely mainly on motorized transportation [76,77], physical education in schools emerges as an essential subject for experiencing and developing health-promoting motor behaviors. Furthermore, a steady increase of studies has demonstrated that motor skills interventions implemented in school settings, outside a physical education setting but still teacher-led, can improve a variety of motor skills in children and adolescents [78]. This has proven especially effective for pupils with motor difficulties or at-risk levels of motor skill [79]. Importantly, these latter sub-groups of children and adolescents also benefit from whole class approaches [80]. Given the results of the current meta-analysis, which clearly demonstrates the positive effects of curriculum-based physical education on the development of motor competence in normally developing children and adolescents, PE as a school subject holds potential for widening the access of resource-efficient support for those experiencing motor challenges in their daily life.

## 5. Conclusions

This aggregate meta-analysis suggests that curriculum-based physical education classes can have a substantial effect on the development of overall motor competence in children and adolescents. To enhance motor competence, teacher-led physical education classes should operationalize a specific curriculum, including e.g., fundamental motor/movement skills, physical literacy and/or gymnastics, which appear to be more effective compared to teacher-led non-specific/standard physical education. Further work should address various factors that might contribute to increased effects of specific physical education content on the development of various dimensions in pupils’ motor competence.

## Figures and Tables

**Table 1:** Chronological overview of studies included in the meta-analysis.

Study	Sample Size (n)	Age (Years)	Total PE (h)	Intervention Group PE	Control Group	Assessment of Motor Competence
Karabouniotis et al. (2002) [44]	45	6	16	Movement skill curriculum	Standard PE curriculum	TGMD
Martin et al. (2009) [45]	54	6	10	High autonomy PE	Low autonomy PE	TGMD-2
Livonen et al. (2011) [46]	84	5	36	Movement literacy curriculum	Standard PE curriculum	FMS
Kalaja et al. (2012) [47]	446	13	50	FMS curriculum	Standard PE curriculum	FMS
Mostafavi et al. (2013) [48]	60	5	*na*	SPARK	Standard PE curriculum	TGMD-2
Teixera Costa et al. (2015) [49]	95	3	36	Structured PE	No PE lessons	PMD
Miller et al. (2016) [50]	107	11	6	Game-centered curriculum	Wait-list	FMS
Jarani et al. (2016) [51]	509	8	30	Exercise or games-based	Standard PE curriculum	KTK
Gallotta et al. (2017) [52]	230	10	40	Fitness or coordination	Standard PE curriculum	KTK
Rudd et al. (2017) [53]	310	8	32	Gymnastics	Standard PE curriculum	TGMD-2, KTK
Rudd et al. (2017) [54]	98	9	16	Gymnastics	Standard PE curriculum	TGMD-2, KTK
Lander et al. (2017) [55]	190	12	18	FMS curriculum	Standard PE curriculum	FMS
Lopes et al. (2017) [56]	60	9	48 and 73	FMS curriculum	No PE lessons	FMS
Gu et al. (2018) [57]	273	11	18	Pedometer-based goal setting	Standard PE curriculum	PE Metrics ^TM^
McGrane et al. (2018) [58]	460	13	37	PA towards health	Standard PE curriculum	TGMD-2
Chan et al. (2019) [59]	276	8	19	AfL + FMS	Standard PE curriculum	TGMD-3
Johnson et al. (2019) [60]	96	4	15	Mastery motivational climate	Standard PE curriculum	TGMD-3
Dalziell et al. (2019) [61]	143	11	32	Better Movers and Thinkers	Standard PE curriculum	FLS
Kriellaars et al. (2019) [62]	211	10	66	Circus arts instruction	Standard PE curriculum	PLAY
Costello and Warne (2020) [63]	100	9	4	Movement literacy	Standard PE curriculum	FMS

Abbreviations: **PE** Physical Education; **MC** Motor Competence; ***na*** Not available; **TGMD** Test of Gross Motor Development; **FMS** Fundamental movement/motor skills; **PLAY** Physical Literacy Assessment for Youth; **PA** Physical Activity; **PMD** Psychomotor developmental profile; **KTK** Körper Koordinationstest Für Kinder; **FLS** Fundamental Locomotor Skills; **AfL** Assessment for Learning; **SPARK** Sports, Play, and Active Recreation for Kids.
